# A thankful recommendation from the Organization for Economic Cooperation and Development to health policy of Japan

**DOI:** 10.1002/jgf2.253

**Published:** 2019-05-20

**Authors:** Yasuharu Tokuda

**Affiliations:** ^1^ Muribushi Okinawa for Teaching Hospitals Okinawa Japan

Medical overuse has become non‐negligible in high‐income countries, including Japan.^1^ False‐positive results from overdiagnosis have led many people to go through downstream examinations and some of them to suffer from complications. Overtreatment causes the increases in side effects of drugs and in complications associated with procedures and surgery.

Overuse of radiation imaging increases the risk of cancer caused by exposure. Japan has the largest number of CT scanners per capita in the world.^2^ There are about 107 machines for every 100 000 people in Japan, that is, almost double the figure in Australia, which ranks second, and greater than four times the Organization for Economic Cooperation and Development (OECD) average of 25 CT machines.

An international campaign to address such low‐value medical care for the people of the world is the Choosing Wisely campaign. It is a call to both patients and healthcare professionals to make better shared decision based on scientific evidence. In the Closing Wisely campaign, about 20 countries and hundreds of clinical societies have agreed and voluntarily make lists of recommendations, typically five list, for excess medical care. In Japan as well,^3^ we have published our own 5‐list recommendations and have formally launched the Choosing Wisely in 2017. However, we have not seen the spread of campaign activities in Japan. Professional clinical societies are expected to voluntarily participate in this campaign.

Recently, the OECD, an international organization that promotes economics and trade among developed countries, has made a proposal to health policy of Japan.^4^ Although the OECD praises Japan's medical care that achieved a long life expectancy, they report that many Japanese people overuse medical tests, especially extensive health checkups program, and that values of these tests should be reviewed. The OECD pointed out Japan's wasteful examinations and unnecessary radiation exposures and suggested that “there is considerable scope for Japan to re‐examine the range of health check‐ups that are in place, evaluating all health check‐ups and cancer screening together, and likely streamlining the range of tests offered.”

The OECD also points out important weakness of preventive medicine that is not conducted in Japan. There is inequality that overlooks the health management of unemployed and part‐time people. For example, many of them do not receive even blood pressure measurement. Blood pressure measuring devices should be placed in spaces such as airports, convenience stores, and parlors.

The OECD is concerned that Japan's smoking rate is still high and that women's alcohol consumption is increasing. Although men's smoking rate shows small decline, it is still around 30% and above the OECD average. Japan should take evidence‐based preventive measures, such as increasing tobacco taxation, strengthening indoor smoking prohibition, and restricting places and time zones where alcohol can be sold. Health policymakers and stakeholders in Japan should use the report as a thankful recommendation for protecting the health of the Japanese people.

## CONFLICT OF INTEREST

The author has stated explicitly that there are no conflicts of interest in connection with this article.
